# Tannic Acid as a Natural Crosslinker for Catalyst-Free Silicone Elastomers From Hydrogen Bonding to Covalent Bonding

**DOI:** 10.3389/fchem.2021.778896

**Published:** 2021-10-18

**Authors:** Sen Kong, Rui Wang, Shengyu Feng, Dengxu Wang

**Affiliations:** ^1^ National Engineering Research Center for Colloidal Materials and Key Laboratory of Special Functional Aggregated Materials, Ministry of Education, School of Chemistry and Chemical Engineering, Shandong University, Jinan, China; ^2^ Shandong Key Laboratory of Advanced Organosilicon Materials and Technologies and State Key Laboratory of Fluorinated Functional Membrane Materials, Zibo, China

**Keywords:** silicone elastomers, tannic acid, catalyst-free, polysiloxane, green chemistry, natural crosslinkers

## Abstract

The construction of silicone elastomers crosslinked by a natural crosslinker under a catalyst-free method is highly desirable. Herein we present catalyst-free silicone elastomers (SEs) by simply introducing tannic acid (TA) as a natural crosslinker when using poly (aminopropylmethylsiloxane-*co*-dimethylsiloxane) (PAPMS) as the base polymer. The crosslinked bonding of these SEs can be easily changed from hydrogen bonding to covalent bonding by altering the curing reaction from room temperature to heating condition. The formability and mechanical properties of the SEs can be tuned by altering various factors, including processing technique, the amount of TA and aminopropyl-terminated polydimethylsiloxane, the molecular weight and -NH_2_ content of PAPMS, and the amount of reinforcing filler. The hydrogen bonding was proved by the reversible crosslinking of the elastomers, which can be gradually dissolved in tetrahydrofuran and re-formed after removing the solvent. The covalent bonding was proved by a model reaction of catechol and *n*-decylamine and occurred through a combination of hydroxylamine reaction and Michael addition reaction. These elastomers exhibit good thermal stability and excellent hydrophobic property and can bond iron sheets to hold the weight of 500 g, indicating their promising as adhesives. These results reveal that TA as a natural product is a suitable “green” crosslinker for the construction of catalyst-free silicone elastomers by a simple crosslinking strategy. Under this strategy, TA and more natural polyphenols could be certainly utilized as crosslinkers to fabricate more organic elastomers by selecting amine-containing polymers and further explore their extensive applications in adhesives, sealants, insulators, sensors, and so forth.

## Introduction

In the past decades, silicone elastomers (SEs) have gained significant attention for extensive applications in aerospace, electronics, protective coating, wearable devices, medical systems, and bio-materials, by virtue of their unique properties, such as high/low temperature resistance, low surface energy, electrical insulation, aging resistance, and biocompatibility (Yilgör and Yilgör, 2014; Rücker and Kümmerer, 2015; Rus and Tolley, 2015; Eduok et al., 2017; Liu et al., 2020). SEs are typically constructed by linking the linear silicone polymers with small molecules or polymers containing two or more linkable sites, which are called crosslinkers, to form three-dimensional (3D) networks. Traditional silicone crosslinking technologies, involving free-radical, condensation and hydrosilylation reactions, have been widely utilized in laboratory and industry (Troegel and Stohrer, 2011; Picard et al., 2015). However, in these processes, catalysts are generally indispensable to accomplish the crosslinking and have brought cost (e.g., platinum catalysts in hydrosilylation) or environmental concerns (e.g., toxic tin catalysts in condensation) (Wang et al., 2017). More importantly, the catalysts that may have an undesirable toxicological effect cannot meet the requirement of green chemistry. Therefore, it is highly desirable to develop a catalyst-free crosslinking strategy.

In recent years, some novel crosslinking strategies, such as azide-alkyne cycloaddition (Rambarran et al., 2012), dynamic covalent bonding (e.g., imine (Bui and Brook, 2020) and boroxine (Lai et al., 2016)), and coordination bonding (Rao et al., 2016; Lai et al., 2018), have been developed to construct silicone elastomers without a catalyst. The key factor in these strategies is to rationally choose a suitable polysiloxane having applicable functional groups as the base polymer. Amine-functionalized polysiloxanes have been verified as a good choice because of their commercial availability and high reactivity, which can be readily crosslinked through versatile catalyst-free reactions, such as aza-Michael reactions (Feng et al., 2016), ureas from reaction with isocyanates (Yang et al., 2020), and imine bonding through reaction with aldehydes (Bui and Brook, 2020). However, in these reactions, the selected crosslinkers are normally synthetic molecules or polymers, thus making it difficult to meet the current requirements of silicone elastomers in terms of green chemistry. Actually, this situation exists in most of silicone materials. Thus it would be of great significance to develop green crosslinkers for silicone elastomers.

Natural polyphenols, featuring more than one phenolic groups, are ubiquitous in nature, such as in fruits, vegetables, grains, tea, coffees, wines, and chocolates, have played a crucial role in defending UV radiation and pathogenic invasion for plants (Xu R. et al., 2018). They not only possess intriguing biological activities (e.g., antioxidant, antiallergic, and antitumor), but also exhibit attractive chemical properties (Xu, et al., 2018a). The abundant phenolic hydroxyl groups in polyphenols can act as donors in the formation of hydrogen bonding. Polyphenols can be easily oxidized to the quinone forms, which can undergo reactions with various functional groups, such as amine and thiol through Michael addition or Schiff base reaction (Wu et al., 2015). The aromatic rings or hydroxyls can also interact with other molecules by hydrophobic or electrostatic interactions. By virtue of these features, natural polyphenols can serve as versatile platforms for material engineering and surface functionalization (Xu L. Q. et al., 2018). In particular, they can be efficiently utilized to link linear polymers together to build crosslinked polymeric materials (Niu et al., 2020), especially high-strength hydrogels (Xu R. et al., 2018; Fan et al., 2018; López and Pich, 2018; Jiménez et al., 2019), and can be recognized as a class of ideal natural and green crosslinkers. However, polyphenols haven’t been utilized as crosslinkers for the construction of elastomers, especially silicone elastomers.

Herein, a typical polyphenol, tannic acid (TA), is chosen as a green and natural crosslinker to fabricate catalyst-free silicone elastomers from amine-functionalized polysiloxanes. By taking the advantage of the feature of hydrogen bond donor from the phenolic hydroxyl group in TA, the elastomers can be readily crosslinked through non-covalent bonds, *i.e.*, hydrogen bond at ambient temperature. It is interesting that the hydrogen bonding can be converted into covalent bonding by improving the crosslinking temperature, thus making TA as a covalent crosslinker for silicone elastomers. Various factors that influence the mechanical properties of these elastomers are systematically investigated. The covalent crosslinking mechanism is explored by designing a model reaction. In addition, the elastomers applied as adhesives were also explored.

## Experimental

### Materials

Tannic acid (TA), catechol, and *n*-decylamine were purchased from Energy Chemical Company of China. Aminopropyl-terminated polydimethylsiloxane (DP-1, M_w_ = 10,000 g mol^−1^) were purchased from Tangui Co., China. All other reagents were purchased from Fuyu Company of China.

### Synthesis of poly (aminopropylmethylsiloxane‐*co*‐dimethylsiloxane) (PAPMS)

**FIGURE 1:**
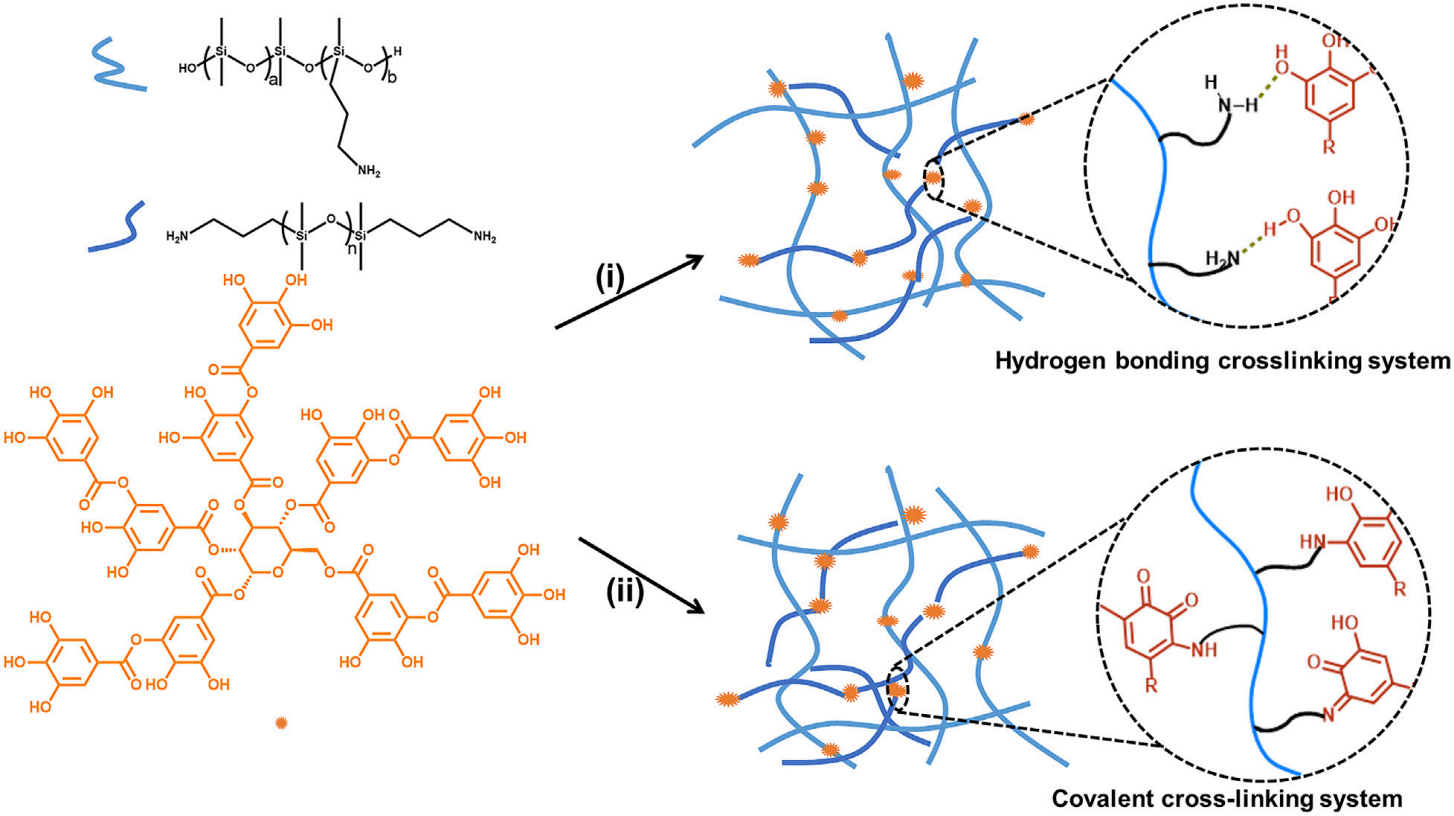
Preparation of silicone elastomers crosslinked by hydrogen bonding or covalent bonding. (i) H_2_O/solvent, room temperature; (ii) H_2_O/solvent, room temperature; 150°C, 1 h.

A series of poly (aminopropylmethylsiloxane-*co*-dimethylsiloxane) (PAPMS) were prepared as our previous report (Lu and Feng, 2017). The structure of PAPMS was shown in Figure 1 and the obtained PAPMS were named as P-X (X represents b/(a+b)× 100, that is, the molar content of–NH_2_ in the main chain), and P-2.2, P-2.5, P-4.8, P-5.8 and P-7.5 were obtained. The molecular weight (M_n_) of these polymers were 1,63,305, 64,000, 45,000, 70,300, and 46,217 g mol^−1^ for P-2.2, P-2.5, P-4.8, P-5.8 and P-7.5, respectively, which were determined by gel permeation chromatography (GPC).

### Preparation of Silicone Elastomers Crosslinked by Hydrogen Bond

In a typical procedure, an aqueous solution of TA was prepared by dissolving TA (30 mg) in water (0.2 ml), while a *n*-hexane solution of amino-containing polysiloxane was prepared by dissolving P-2.5 (1.6 g) and aminopropyl-terminated polysiloxane (DP-1) (0.6 g) in *n*-hexane (15 ml). Then the organic solution and TA aqueous solution were mixed evenly and poured into a Teflon mold. After 24 h, a transparent and dark yellow silicone elastomer was obtained. Table 1 summarizes the variables for the synthesis of SEs (H-1 to H-16) crosslinked by hydrogen bond.

**TABLE 1:** Data of hydrogen bond cross-linked silicone elastomers.

Entry	PAPMS (g)	DP-1 (g)	TA (mg)	Solvents/H_2_O (ml)^a^	Sample description
Solvent Type					
H-1	P-2.5 (1.6)	0.6	30	TOL (15/0.2)	Formable
H-2	P-2.5 (1.6)	0.6	30	HEX (15/0.2)	Formable
H-3	P-2.5 (1.6)	0.6	30	THF (15/0.2)	TA aggregation
H-4	P-2.5 (1.6)	0.6	30	DCM (15/0.2)	×
H-5	P-2.5 (1.6)	0.6	30	TCM(15/0.2)	×
DP-1 dosage/-NH_2_ molar content					
H-6	P-2.5 (1.6)	0	60	HEX (15/0.3)	Formable
H-7	P-2.5 (1.6)	0.6	60	HEX (15/0.3)	Formable
H-8	P-2.5 (1.6)	0.9	60	HEX (15/0.3)	×
H-9	P-5.8 (1.6)	0.6	60	HEX (15/0.3)	Formable
TA dosage					
H-10	P-2.5 (1.6)	0.6	8	HEX (15/0.2)	×
H-11	P-2.5 (1.6)	0.6	30	HEX (15/0.2)	Formable
H-12	P-2.5 (1.6)	0.6	50	HEX (15/0.3)	Formable
H-13	P-2.5 (1.6)	0.6	60	HEX (15/0.3)	Formable
H-14	P-2.5 (1.6)	0.6	90	HEX (15/0.4)	×
Molecular weight change (P-2.2: M_n_ = 1,63,305 g mol^−1^, P-2.5: M_n_ = 64,000 g mol^−1^)					
H-15	P-2.2 (1.6)	0.6	50	HEX (20/0.3)	Formable
H-16	P-2.5 (1.6)	0.6	50	HEX (20/0.3)	Formable

a THF, tetrahydrofuran; DCM, dichloromethane; TCM, trichloromethane; HEX, n-hexane; TOL, toluene.

To improve the mechanical properties of the hydrogen bonding SEs, the reinforcing filler was added. Taking H-17 as an example, 0.22 g of fumed silica H2000 was added to DP-1 (0.6 g) and P-2.5 (1.6 g) in *n*-hexane solution, and then TA in aqueous solution (30 mg of TA in 0.2 ml of water) was added and mixed with the organic solution. Finally, the mixture was poured into the Teflon mold and remained at room temperature. After 24 h, a transparent silicone elastomer was obtained. Supplementary Table S2 summarizes the formulation data for hydrogen-bonding elastomers after the reinforcement.

### Preparation of Silicone Elastomers Crosslinked by Covalent Bond

In a typical procedure, TA (30 mg) was dissolved in 0.3 ml of water, forming an aqueous solution of TA, while DP-1 (0.6 g) and P-5.8 (1.6 g) were dissolved in *n*-hexane. Then the TA solution and hexane solution were mixed evenly. The resulting mixture was poured into a Teflon mold. After evaporating the solvents at room temperature (∼24 h), the resultant film was heated at 150°C for 1 h and a transparent and brown silicone elastomer was obtained. Supplementary Table S1 summarizes the variables for the synthesis of SEs (C-1 to C-17) crosslinked by covalent bond.

The reinforcing filler was also added to improve the mechanical property. Taking C-18 as an example (Supplementary Table S2), fumed silica H2000 (0.22 g) was added into *n*-hexane solution containing (DP-1) (0.6 g) and P-2.5 (1.6 g). Then TA in aqueous solution was added to mixture and stirred rapidly for ca. 15–20 min. The resultant mixture was poured into a Teflon mold and remained at room temperature to remove the solvents (ca. 24 h), affording a film. The film was heated at 150°C for 1 h and the final silicone elastomer was obtained. Supplementary Table S2 summarizes the formulation data for covalently bonding elastomers after the reinforcement.

### Measurement of Crosslinking Density

The average molecular weight (M_c_) between the crosslinking points is usually used to indicate the crosslinking density of silicone elastomers. It is generally measured using an equilibrium swelling method in toluene (Lu and Feng, 2017). The silicone elastomers were dried before measurement and weighed as mass m_1._ Then the silicone elastomer was immersed in toluene at room temperature (ca. 3 days). The swollen gel was taken out from the liquid when the weight of the gel (m_2_) kept constant (excess solvent was removed from the surface). The volume fraction Φ of the silicone elastomer dissolved in toluene was calculated by Eq. 1.

$$\Phi =\frac{\frac{m_{1}}{\rho_{2}}}{\frac{m_{2}-m_{1}}{\rho_{1}}+\frac{m_{1}}{\rho_{2}}}$$ (1)

m_1_ and m_2_ represent the masses of silicone elastomers before and after swollen in toluene, respectively. ρ_1_ and ρ_2_ represent the densities of toluene and silicone elastomers, respectively. M_c_ was calculated by Flory and French’s Eq. 2. V_1_ and χ represent the molar volume of toluene and the Flory-Huggins interaction parameter of polysiloxane and toluene (0.465).

$$M_{c}=-\frac{\rho_{2\;}V_{1}{\text{Φ}}^{\frac{1}{3}}}{\ln\left(1-\text{Φ}\right)+\text{Φ}+\chi {\text{Φ}}^{2}}$$ (2)

The crosslink density ν can be calculated by Eq. 3.

$$\text{ν}=\frac{\rho_{1}}{M_{c}}$$ (3)

### Model Reaction of Catechol and *n*-decylamine

In a flask, catechol (0.5515 g, 5 mmol) and *n*-decylamine (1.5729 g, 10 mmol) were added and mixed. The mixture was heated at 150°C for 1 h under stirring. After the reaction finished, the product was afforded as a brown liquid.

### Application as Adhesives for Bonding Iron Sheets

Taking B-3 as an example, TA (15 mg) and PAMPMS (0.4 g) was mixed in *n*-hexane (5 ml). The mixture was coated evenly on two 2 × 6 cm iron sheets and the bonding method is the lap joint. The application method and test method are performed in accordance with the national standard (GB 7124–2008). With the adhesive crosslinked by hydrogen bond, the bonded iron sheets were placed at room temperature to remove the solvent (24 h) before testing. With the adhesives crosslinked by covalent bond, the bonded iron sheets were heated at 150°C for 1 h before testing. Supplementary Table S4 summarizes the formulation of adhesives.

## Results and Discussion

### Synthesis and Characterization

Novel silicone elastomers were prepared using aminopropyl functionalized polysiloxanes as the base polymers and TA as the crosslinker at different conditions (Figure 1). At room temperature, the silicone elastomers, H-1 to H-16, were crosslinked by hydrogen bond between amine groups from the polysiloxanes and phenolic groups from TA. After heating the hydrogen bonding elastomers at 150°C for 1 h, the silicone elastomers, C-1 to C-17, crosslinked by covalent bond were obtained.

**FIGURE 2:**
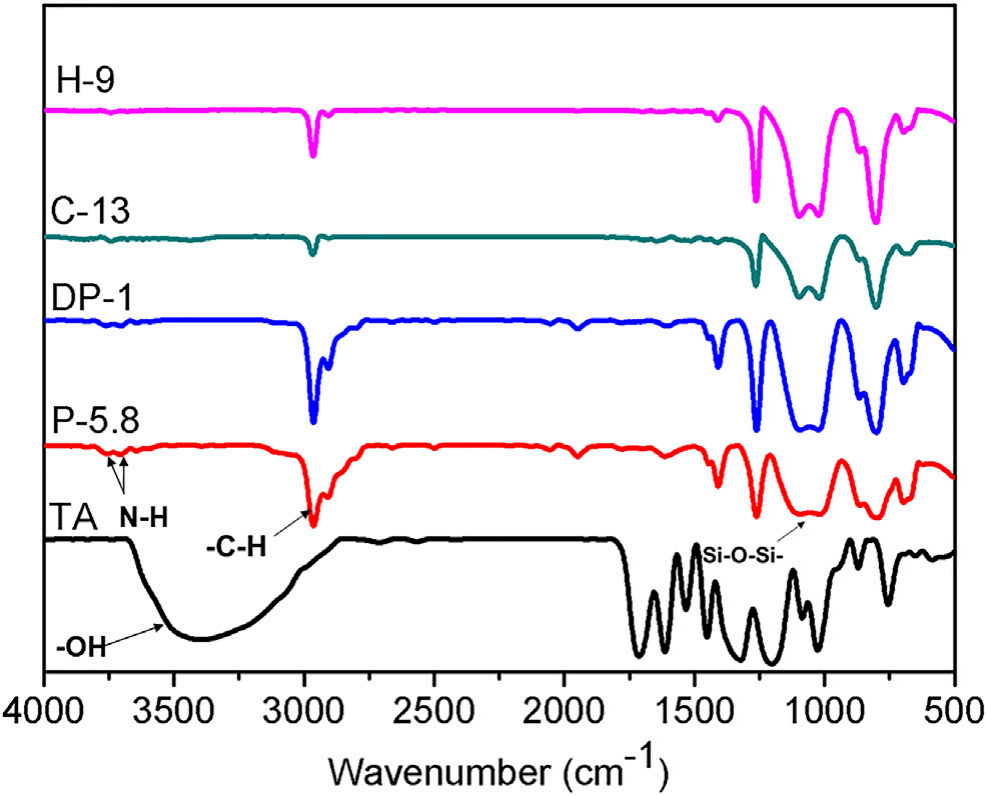
FT-IR spectra of TA, P-5.8, DP-1, C-13, and H-9

The formed silicone elastomers were characterized by FT-IR technique. Figure 2 shows the IR spectra of raw materials (P-5.8, DP-1 and TA) and elastomers (H-9 and C-13 as examples). The characteristic absorption of phenolic groups are observed at 3,450 cm^−1^ in TA, while the characteristic absorption peak of -N-H bond in amine groups from DP-1 and P-5.8 are observed at 3,700 cm^−1^ and 3,650 cm^−1^. After crosslinking, these peaks nearly disappear in the elastomers (H-9 and C-13), thereby indicating that the interaction or a reaction between phenolic and amine groups occurred. In addition, the strong absorption peaks in the range of 1,022–1,094 cm^−1^ in P-5.8, DP-1, and C-13 are attributed to the characteristic Si-O-Si stretching vibration.

### Effect of Variables on the Properties of Hydrogen Bond Crosslinked SEs

As mentioned above, by virtue of the abundant phenolic groups in TA, it is expected to crosslink amino-functionalized polysiloxanes *via* the hydrogen bonding between phenolic hydroxyl and amine groups (Figure 1). To fabricate a high-value SE, it is indispensable to investigate the various effects on the formability and mechanical properties of the SEs. Table 1 summarizes the variables that were examined to evaluate the formability of silicone elastomers based on hydrogen bonding.

The first factor is the processing technique. It is known that polysiloxanes containing low content of aminopropyl groups are insoluble in water, while TA is merely soluble in water. This feature makes TA difficult to be evenly dispersed in the polysiloxane system. Thus the crosslinking process was performed in a mixed solvent. In other word, the crosslinking proceeded by adding the aqueous solution of TA into the organic solution of aminopropyl polysiloxane. It is found that when the organic solvent was dichloromethane (H-4) or trichloromethane (H-5), the elastomers could not be formed. This finding can be explained by the phenomenon that dichloromethane and trichloromethane evaporate too quickly, resulting in the inability of tannic acid and PMPMS to form an effective contact. Although the crosslinking can occur in tetrahydrofuran (H-3), TA aggregated in the formed film. It is delighted that the SEs can be well-formable when using toluene (H-1) or *n*-hexane (H-2) as the solvent. Considering the toxicity of toluene, *n*-hexane was chosen as the organic solvent for the preparation of SEs. In addition, we found that if the solvent is insufficient, the formation of hydrogen bond in the system led to an increase of the viscosity, which made the elastomers be formed with some defects, *i.e.*, some bubbles in the elastomers. Thus, more solvent is required during the crosslinking process to reduce the viscosity of the system. It was found that the suitable volumes of solvent and water are 15 and 0.2 ml while the selected parameters include 1.6 g of P-2.5, 0.6 g of DP-1, and 30 mg of TA.

**FIGURE 3:**
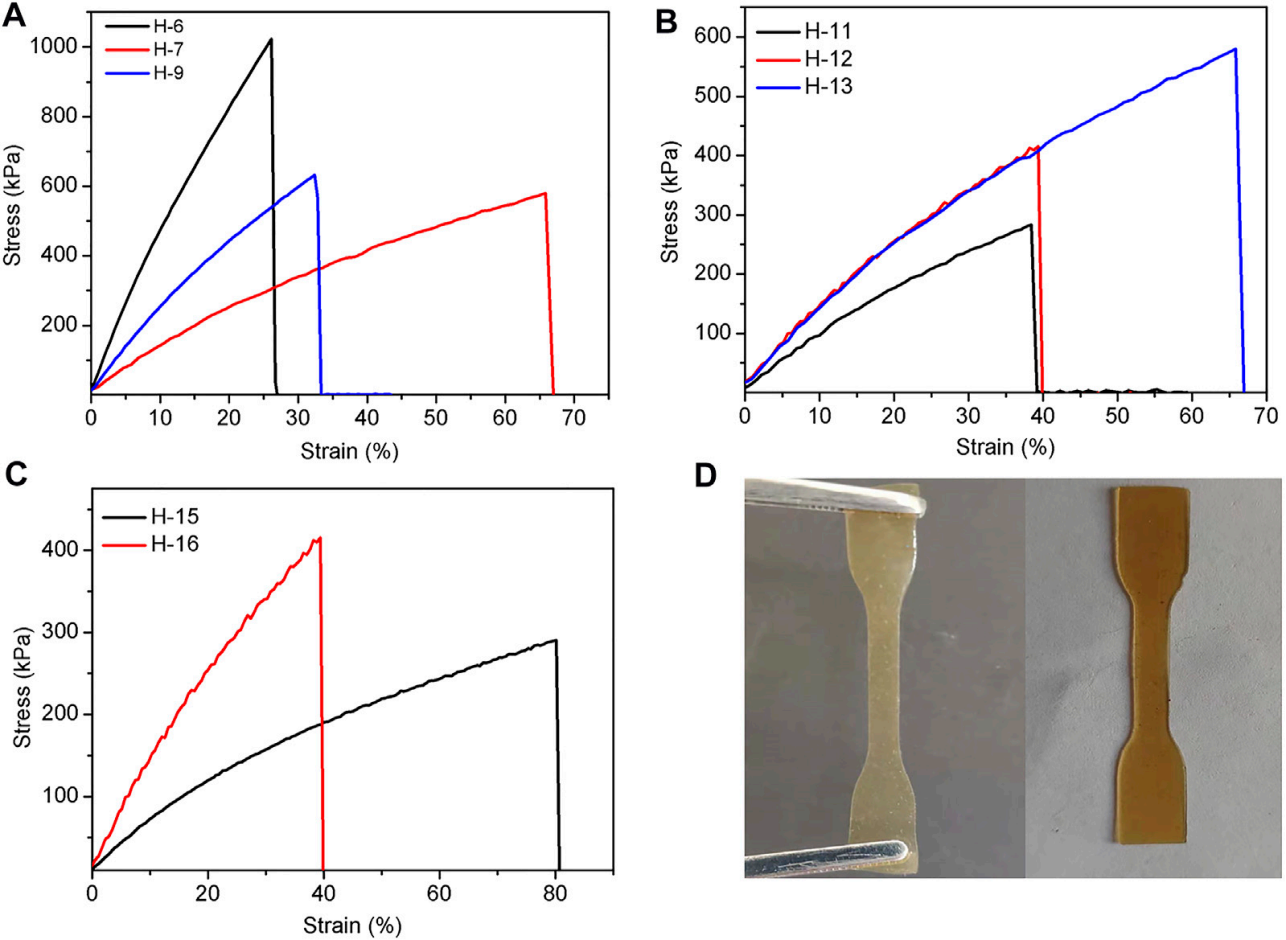
**(A–C)** Tensile curves of hydrogen bond cross-linked silicone elastomers affected by amino content of PAPMS and the usage of DP-1 **(A)**, TA dosage **(B)**, and molecular weight of PAPMS **(C)**; **(D)** The images of H-11 **(left)** and H-13 **(right)**.

It is known that the addition of a base polymer with terminated functional group can enhance the break elongation of elastomers. Thus, amino-terminated polysiloxane DP-1 was added to study this effect. When the elastomer was cross-linked by hydrogen bond, we added no DP-1 in H-6 and 0.6 g of DP-1 in H-7. It was found that the tensile strength of the SEs decreased from 1,023 kPa (H-6) to 580 kPa (H-7) (Figure 3A). However, the elongation at break increased from 26% (H-6) to 66% (H-7). This phenomenon suggests that the addition of DP-1 can slightly increase the elongation at break of the elastomers. However, the Young’s modulus and cross-link density decreased in a certain. This finding may be attributed to the low reactivity of DP-1, which can hinder the crosslinking.

The mechanical properties of hydrogen bond cross-linked elastomers are also affected by the -NH_2_ content in PAPMS. As shown in Figure 3A, when the molar content of -NH_2_ in PAPMS was increased from 2.5% (H-7) to 5.8% (H-9), the tensile strength increased from 580 kPa (H-7) to 632 kPa (H-9), but the elongation at break decreased from 66% (H-7) to 32% (H-9). This finding is apparently due to the enhancement of crosslinking density (0.16–0.32 mol/L) and Young’s modulus (1.4–2.5 MPa), which was led by the increased -NH_2_ contents (Table 2). The amount of the crosslinker TA undoubtedly influences the mechanical properties of SEs. As expected, too many or too few amounts of TA is detrimental to the formation of elastomers. For example, if the amount of TA is 8 mg (H-10), the elastomer cannot be formed due to too few crosslinking sites (The other parameters include 1.6 g of P-2.5, 0.6 g of DP-1, and 15/0.2 ml of hexane/water). When the amount of TA is too high, such as 90 mg (H-14), the cross-linking rate is too fast, resulting in significant defects in the elastomer. It was found that the TA dosages from 30 to 60 mg are suitable and the elastomers (H-11, H-12, and H-13) were obtained with good formability. As shown in Figure 3B, with an increment of TA dosages, the tensile strength was enhanced from 283 kPa (H-11) to 580 kPa (H-13), and the elongation at break and Young’s modulus also increased from 38% (H-11) to 66% (H-13) and from 1.0 MPa (H-11) to 1.4 MPa (H-13), respectively (Table 2). This finding indicates an increase in the material’s ability to resist deformation with increasing the amount of TA. In addition, the colors of the resultant elastomers turned from pale yellow to brownish-yellow (H-11 and H-13 in Figure 3D).

**TABLE 2:** Mechanical properties of hydrogen bond cross-linked SEs.

Entry	Tensile strength (kPa)	Elongation at break (%)	Hardness (shore A)	Young’s modulus (MPa)	Crosslink density (mol/L)
H-6	1,023	26	34.0	5.1	0.25
H-7	580	66	33.1	1.4	0.16
H-9	632	32	31.0	2.5	0.32
H-11	283	38	24.0	1.0	0.16
H-12	415	39	31.8	1.4	0.16
H-13	580	66	33.1	1.4	0.16
H-15	290	80	30.5	0.6	0.14
H-16	415	39	31.8	1.4	0.16

The molecular weights of PAPMS can also determine the properties of the elastomers. Two PAPMS with M_n_ of 163,305 g mol^−1^ (P-2.2) and 64,000 g mol^−1^ (P-2.5) acted as the base polymers when the other parameters include 0.6 g of DP-1 and 20/0.4 ml of hexane/water (*Note*: it is difficult to precisely control the molecular weight and the–NH_2_ content of the PAPMS). Due to the large molecular weight of P-2.2, 50 mg of tannic acid was added at room temperature for effective crosslinking. The results reveal that PAPMS with higher molecular weight can give the elastomers with higher elongation at break (Figure 3C), but lower tensile strength (H-15 and H-16). This result can be explained by the different crosslinking density. Although a higher molecular weight can lead to a better ductility of the cross-linked network, but higher molecular weight of PAPMS at the same mass means a lower amount of amino groups, that is, fewer crosslinking sites, thereby leading to a lower degree of cross-linking density. This result was also evidenced by the Young’s modulus (Table 2).

### Effect of Variables on the Properties of Covalent Bond Crosslinked Silicone Elastomers

**FIGURE 4:**
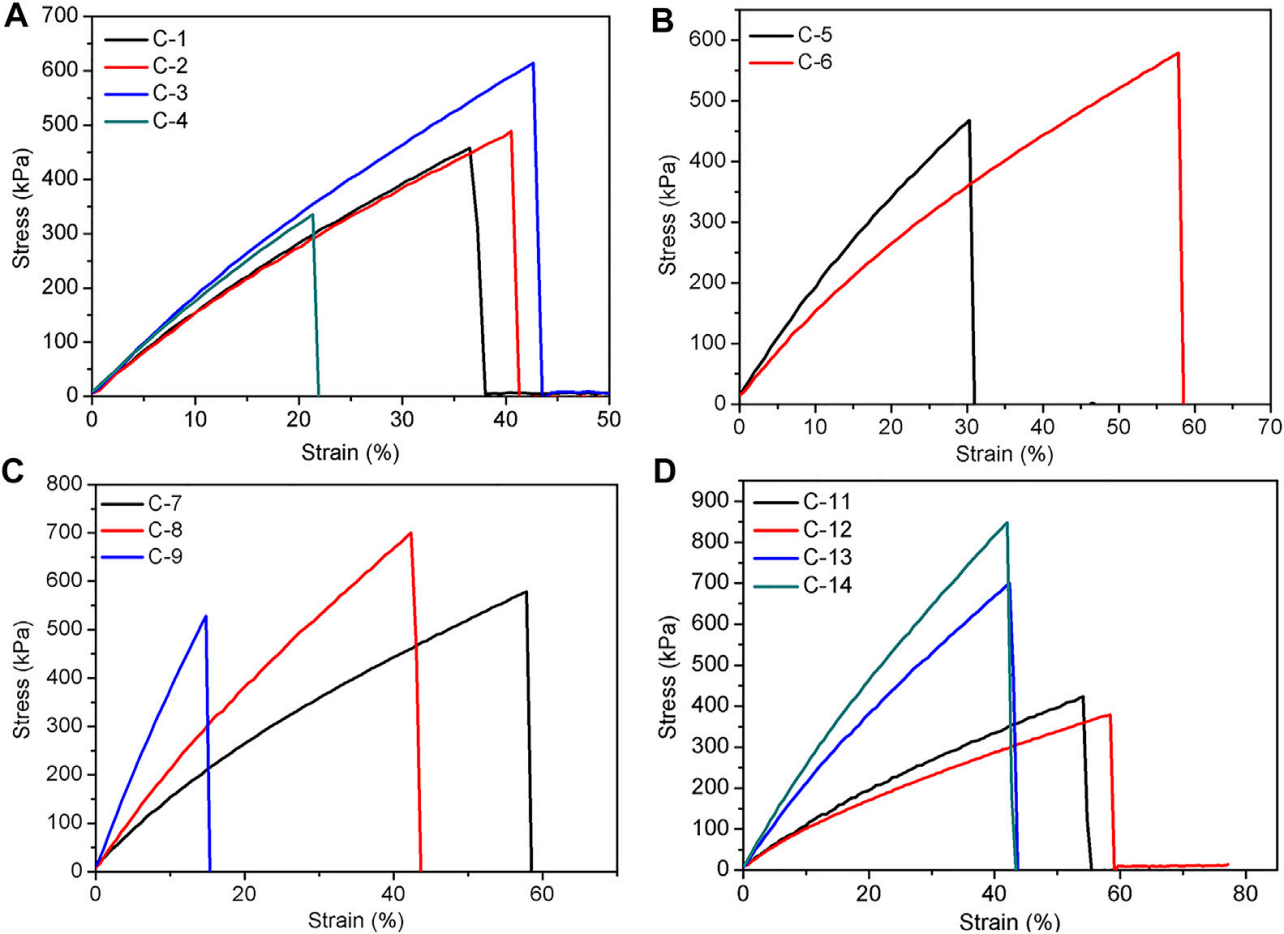
The tensile curves of covalently bonded elastomers influenced by heating time **(A)**, DP-1 **(B)**, -NH_2_ content **(C)**, and TA content **(D)**.

As mentioned above, the covalently crosslinked SEs can be readily prepared by heating the hydrogen bonding SEs at 150°C. The effect of various factors on the formability and mechanical properties of elastomers were also investigated and were summarized in Supplementary Table S1 and Table 3. The first variable is the heating time. When the selected parameters include 1.6 g of P-4.8, 0.6 g of DP-1, 30 mg of TA, and 15/0.2 ml of hexane/water, with an increment of heating time from 1 to 3 h, the tensile strength of the elastomer increased from 458 kPa (C-1) to 614 kPa (C-3), and the elongation at break increased from 36% (C-1) to 43% (C-3) (Figure 4A). This phenomenon suggests that the mechanical properties of elastomers can be enhanced by prolonging the heating time. Meanwhile, Young’s modulus increased from 1.6 to 1.9 MPa and crosslink density increased from 0.22 to 0.44 mol/L (Table 3). However, as the heating time continued to increase to 4 h, a decrease of the mechanical property of the elastomer (C-4) was found. This finding may be due to the fact that excessive cross-linking occurs within the networks due to prolonged heating.

**TABLE 3:** Mechanical properties of covalent cross-linked silicone elastomers.

Entry	Tensile strength (kPa)	Elongation at break (%)	Hardness (shore A)	Young’s modulus (MPa)	Crosslink density (mol/L)
C-1	458	36	26.4	1.6	0.22
C-2	489	41	22.0	1.6	0.36
C-3	614	43	22.4	1.9	0.44
C-4	336	21	×	1.7	0.32
C-5	468	30	31.8	1.9	0.42
C-7	579	58	47.4	1.4	0.15
C-8	700	42	30.0	2.2	0.47
C-9	528	15	20.2	3.8	0.68
C-11	425	54	25.4	1.1	0.28
C-12	503	72	27.7	1.0	0.35
C-13	700	42	30.0	2.2	0.47
C-14	848	42	38.3	2.6	0.80
C-16	249	27	28.3	1.1	0.15
C-17	199	67	×	0.4	0.12

× means the sample is not available for the test.

Amino-terminated polysiloxane DP-1 was used as a chain extender for the cross-linking of elastomers. The results showed that the elastomer (C-5) had a tensile strength of 468 kPa and an elongation at break of 30% without the addition of DP-1 (Figure 4B). When 0.6 g of DP-1 was added, the tensile strength of the elastomer (C-7) was 579 kPa and the elongation at break was 58% (Figure 3C). It can be seen that the addition of DP-1 can improve the mechanical properties of the elastomers. At the same time, excessive DP-1 is not conducive to the formation of cross-linked networks, and this phenomenon is similar to that of hydrogen bonding system. The Young’s modulus and crosslink density of the elastomer underwent a decrease due to the addition of DP-1 (Table 3). In addition, the effect of -NH_2_ content in PAPMS is similar to that of hydrogen bond cross-linked system (Figure 4C). As the -NH_2_ content increases from 2.5 to 5.8 mol%, the tensile strength increases from 579 kPa (C-7) to 700 kPa (C-8) and the elongation at break largely decreases from 58% (C-7) to 42% (C-8) (Figure 4C) (Table 3). This finding can be explained as follows. As the amino content of PAPMS increases, the cross-linking degree of the elastomers increases and their mechanical properties are improved. This explanation can be also evidenced by the Young’s modulus and crosslink density (Table 3). However, as the amino content continues to increase, the elastomer (C-9) becomes excessively cross-linked and thus leads to a decrease in tensile strength and elongation at break.

**FIGURE 5:**
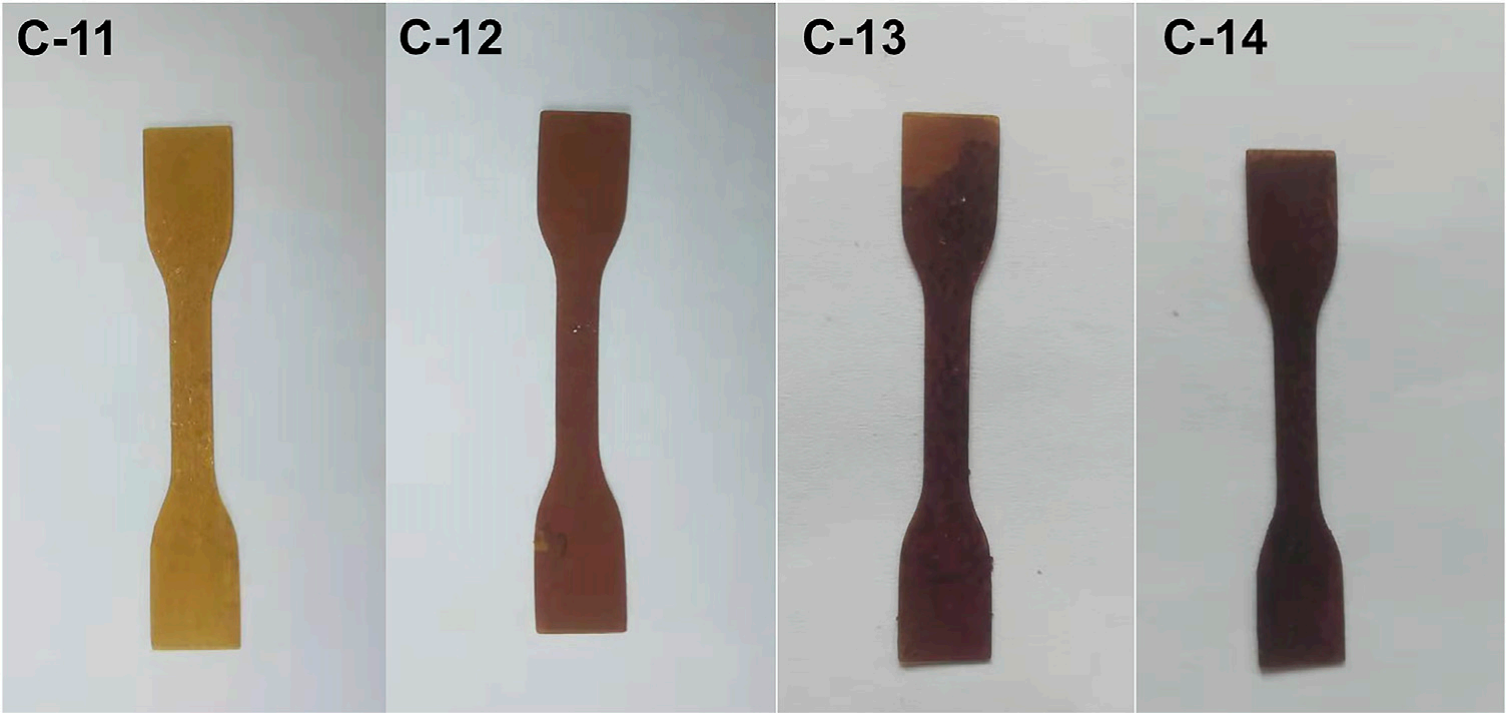
Images of covalent bond crosslinked elastomers by altering the dosage of TA.

Based on the abovementioned results, the elastomers with the best elastomeric properties were prepared from P-5.8. Thus it was utilized as the base polymer to study the effect of TA dosage on the elastomer properties. As expected, if the amount of TA is too low (e.g., 8 mg for C-10 when the other parameters include 1.6 g of P-5.8, 0.6 g of DP-1, and 15/0.2 ml of hexane/water), the elastomer can not be formed due to the low crosslinking density. If the amount of TA is too high (e.g., 120 mg for C-15), the elastomer can be formed, but appeared as a film with apparent defect on the bottom because of too fast crosslinking rate. It was found that suitable amounts of TA are in the range of 10–90 mg. With an increment of TA dosage, the tensile strength increases from 425 kPa (C-11) to 848 kPa (C-14), while the elongation at break of the elastomers slightly decreases from 54% (C-11) to 42% (C-14) (Figure 4D). This finding is apparently due to the improved crosslinking density from 0.28 mol/L (C-11) to 0.80 mol/L (C-14) and Young’s modulus increased from 1.1 MPa (C-11) to 2.6 MPa (C-14) as increasing the TA dosage (Table 3). This result indicates that the increase of TA dosage can improve the mechanical properties of the elastomers. Meanwhile, the hardness also increased from 25.4 Shore A (C-11) to 38.4 Shore A (C-14) (Table 3). It can be seen that in the covalent bonding cross-linking system, the increase of TA dosage can increase the hardness of the elastomers and has a reinforcing effect (Zhang et al., 2015). In addition, the increment of TA dosage makes the colors of products darken from deep yellow to nearly black (Figure 5).

The effect of molecular weight of PAPMS on the mechanical properties of silicone elastomers is similar to that found in hydrogen bond crosslinked SEs. P-2.2 (M_n_ = 163,305) and P-2.5 (M_n_ = 64,000) were chosen as the base polymers to investigate the effect of molecular weight (other parameter include 0.6 g of DP-1, 30 mg of TA, and 15/0.3 ml of hexane/water). As shown in Supplementary Figure S1, the elongation at break of the elastomers decreases from 249 kPa (C-16) to 199 kPa (C-17) with increasing the molecular weight, and the elongation at break increases from 27% (C-16) to 67.4% (C-17). Meanwhile, Young’s modulus and crosslinking density decrease to some degree (Table 3). This result can be explained by the fact that higher molecular weight of the base polymer means less crosslinking sites and thus the crosslinking degree is low, but the elongation at break of the elastomer can increase.

### The Performance Enhancement of SEs by Adding Reinforcing Fillers

**FIGURE 6:**
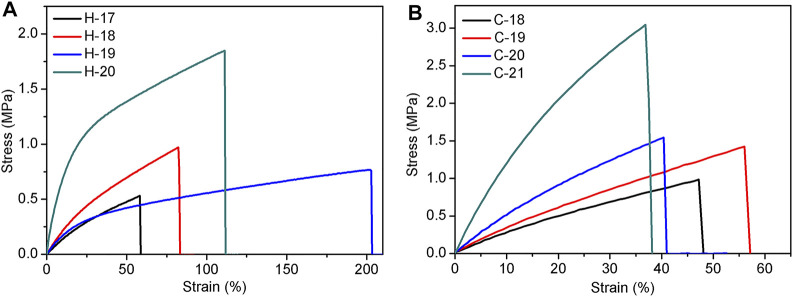
The tensile curves of hydrogen bond cross-linked silicone elastomers, H-17 to H-20 **(A)** and covalent bond crosslinked silicone elastomers, C-18 to C-21 **(B)**, after adding the reinforcing filler.

Based on the abovementioned results, the mechanical properties of these elastomers crosslinked either by hydrogen bond or by covalent bond are not satisfied. To improve elastomer properties, the reinforcing filler was added and the fumed silica H2000 was used as an example. The formability and mechanical data of the resultant SEs are summarized in Supplementary Tables S2, S3. As expected, the mechanical properties of the elastomers, H-17 to H-20, were gradually improved as the amount of H2000 increased. In the hydrogen bond crosslinked system, the maximum tensile strength is 1.9 MPa (H-20), while the elongation at break of this elastomer is 111% (Figure 6A, the parameters include 1.6 g of P-2.5, 0.6 g of DP-1, and 15/0.2 ml of hexane/water). When the amount of H2000 continues to increase, the mechanical properties of the elastomer (H-21) will decline because too much H2000 hinders the formation of the cross-linked network. These hydrogen bond cross-linked elastomers (H-17 to H-20) were further heated at 150°C for 1 h to yield covalently cross-linked elastomers, C-18 to C-21. As shown in Figure 6B, the tensile strength was further increased to 3.0 MPa at a dosage of 0.88 g of H2000 (C-21). Compared to the analogous hydrogen bond crosslinked elastomer H-20, the tensile strength was increased by 50%. This finding can be explained by the increased cross-linking density (e.g., 0.45 mol/L for H-20 to 1.23 mol/L for C-21) after the transformation from hydrogen bond crosslinking to covalent crosslinking. In addition, the Young’s modulus increased with the amount of H2000 in both the hydrogen bonding and covalent bonding cross-linking systems. In other word, the crosslinking density of the elastomer formed by the covalent system is greater than that of the elastomer formed by the hydrogen bonding system under the same formulation (Supplementary Table S3).

### Cross-Linking Mechanism

**FIGURE 7:**
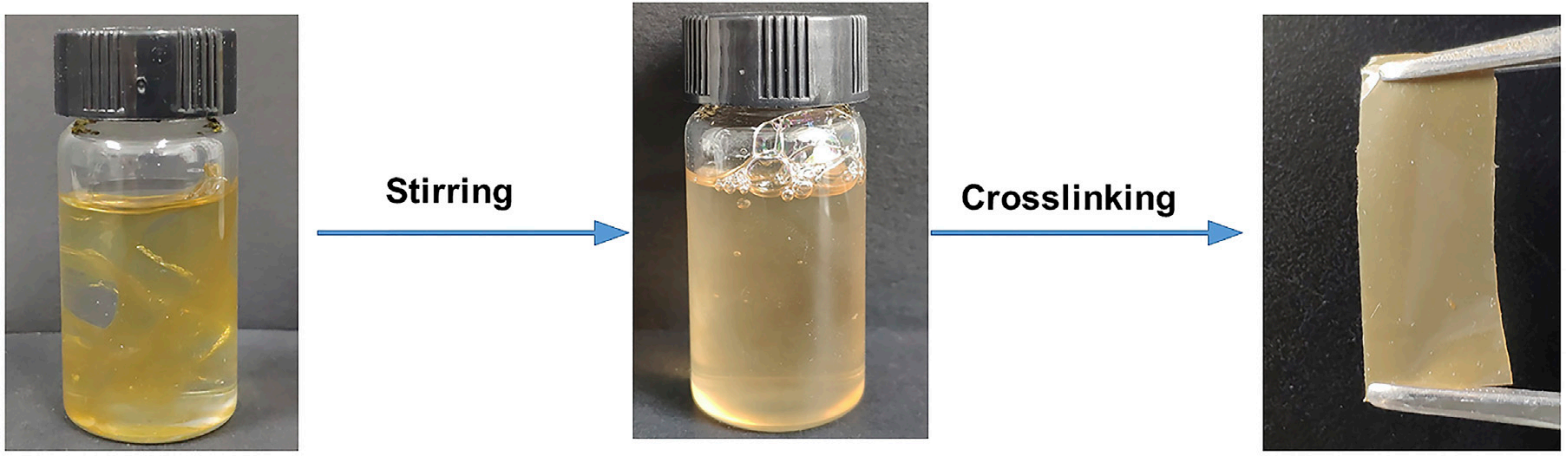
The reversible film formation by hydrogen bonding crosslinking.

The hydrogen bond crosslinking mechanism was investigated by immersing the elastomers in tetrahydrofuran (THF), which can serve as a hydrogen bond acceptor and thus interrupt the hydrogen bond crosslinking. It was found that the elastomers can be gradually soluble in THF under stirring and re-formed after removing the solvent (Figure 7). This reversible crosslinking feature accords with the characteristic of hydrogen bond, indicating the networks are formed by hydrogen bond crosslinking.

**FIGURE 8:**
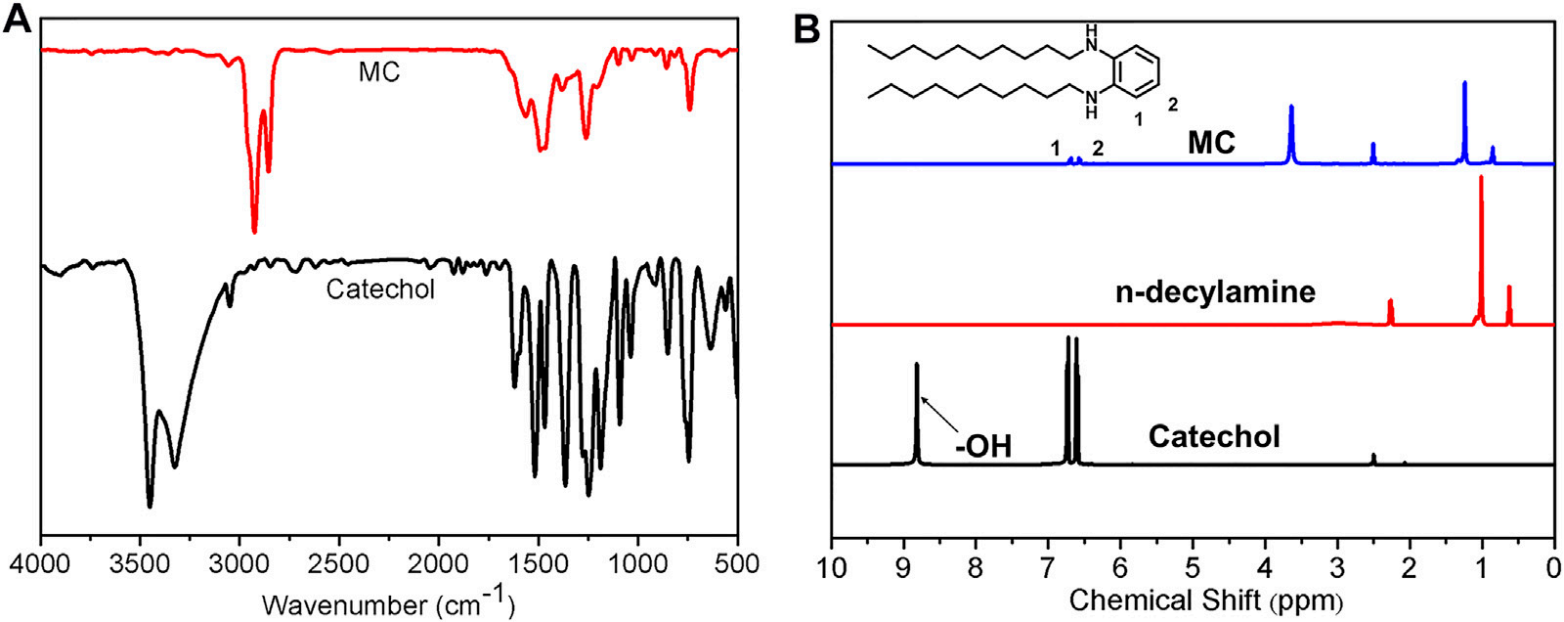
**(A)** FT-IR spectra of catechol and MC; **(B)** ^1^H NMR spectra of catechol, *n*-decylamine and MC.

The cross-linking mechanism by covalent bond between TA and PAPMS was investigated by a model reaction between catechol and *n*-decylamine (Supplementary Figure S2). The reaction condition was 1 h at 150°C, resulting in the products MC. The products were characterized by FT-IR, ^1^H NMR and high resolution mass spectrometry. In FT-IR spectrum of catechol and the products, the broad characteristic peaks of–OH from catechol was observed at 3,500 cm^−1^ and 3,300 cm^−1^ and disappeared in MC (Figure 8A). This finding indicates the successful occurrence of some reactions, for example, hydroxylamine reaction, between catechol and *n*-decylamine. The ^1^H NMR spectra also proves this result (Figure 8B). The proton peak from the phenolic hydroxyl group in catechol was observed at 8.8 ppm, while this peak disappeared in the product MC, indicating the reaction of the phenolic hydroxyl group. In addition, the protons from the benzene rings shift from 6.72 to 6.61 ppm in catechol to around 6.68–6.57 ppm in MC, further indicating that phenol was not fully oxidized and the hydroxylamine reaction occurred. The HR-MS results can prove the structure of the products. As shown in Supplementary Figure S3, the found weights of 250.2112, 406.3686, and 558.5202 g/mol are associated with the products by the hydroxylamine reaction of amino and–OH groups in TA and Michael addition reaction of amine and the quinone groups after the oxidation of TA, respectively, consistent with previous results (Liang et al., 2016). In addition, by comparing the ^1^H NMR of the mixture of catechol and *n*-decylamine before the reaction and MC, ca. 70% of catechol underwent a hydroxylamine reaction and the rest proceeded by the oxidation reaction of phenolic group and subsequent Michael addition reaction, which was calculated on the basis of integration ratio of the alkyl protons from *n*-decylamine and the phenyl protons from catechol (Supplementary Figure S4). These results reveal that the covalent crosslinking of SEs is determined by the combined contribution of the hydroxylamine reaction and Michael addition reaction, while the former is the main contributor.

### Elastomer Curing Time Analysis

**FIGURE 9:**
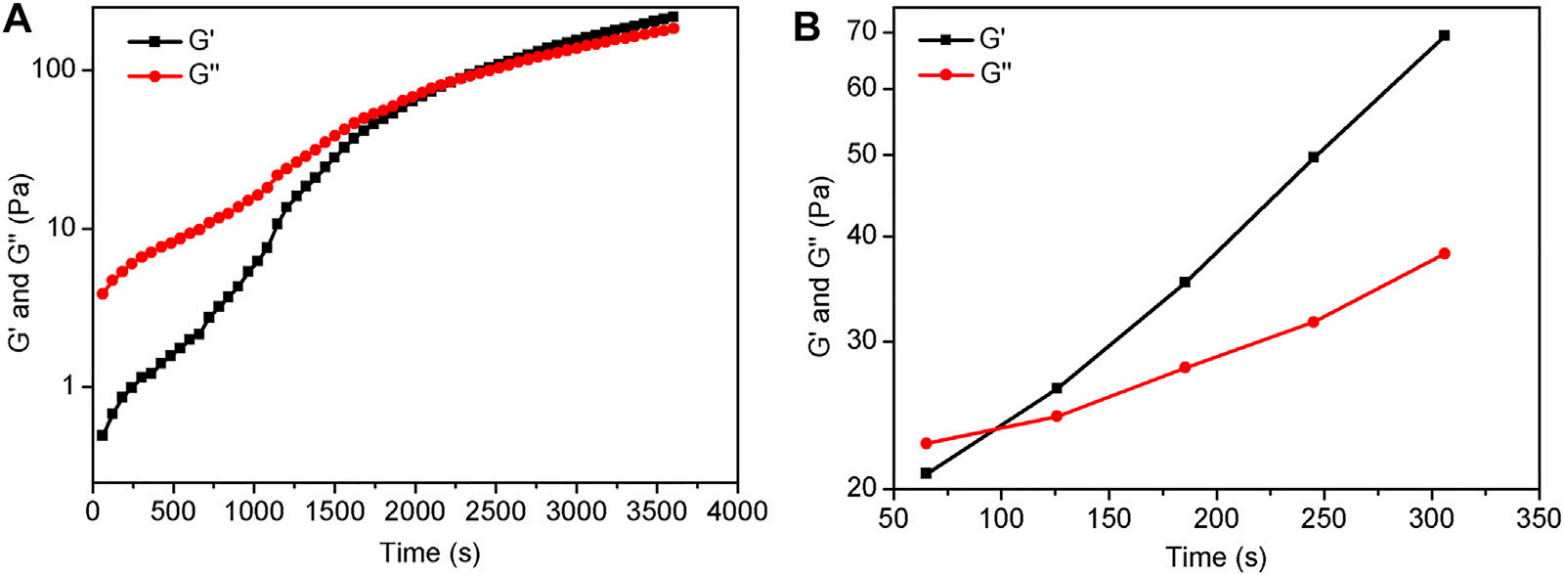
Rheological curves of H-9 **(A)** and C-13 **(B)** s.

To investigate the specific crosslinking time between TA and aminopropyl polysiloxane, the rheometer was used to analyze the curing time of these two cross-linked systems. The hydrogen bond crosslinking system was studied using H-9 as an example. A representative parameters include P-5.8 (1.6 g), DP-1 (0.6 g), TA (60 mg), and *n*-hexane/water (15/0.3 ml). Figure 9A shows the curves of loss modulus (G″) and energy storage modulus (G′) of the hydrogen bond crosslinking system with curing time. It was found that G″ is larger than G′ before 37 min, indicating that the system did not cure. The intersection point of G″ and G′ is the gel-solution transition point. As the time increases, G′ becomes larger and the system becomes a gel. The curing behavior of covalent bond cross-linking system was investigated using C-13 as an example, which was tested at 150°C. It was found that the G″ and G′ crossover point is about 90 s (Figure 9B). The difference in time between these two crosslinking systems is clearly related to the reaction conditions. The hydrogen bonding crosslinking and the covalent bonding crosslinking occurs at room temperature and at 150°C, respectively. Obviously, higher temperature resulted in higher reactivity, and thus in faster curing time.

### Micromorphology and Hydrophobic Properties of Silicone Elastomers

**FIGURE 10:**
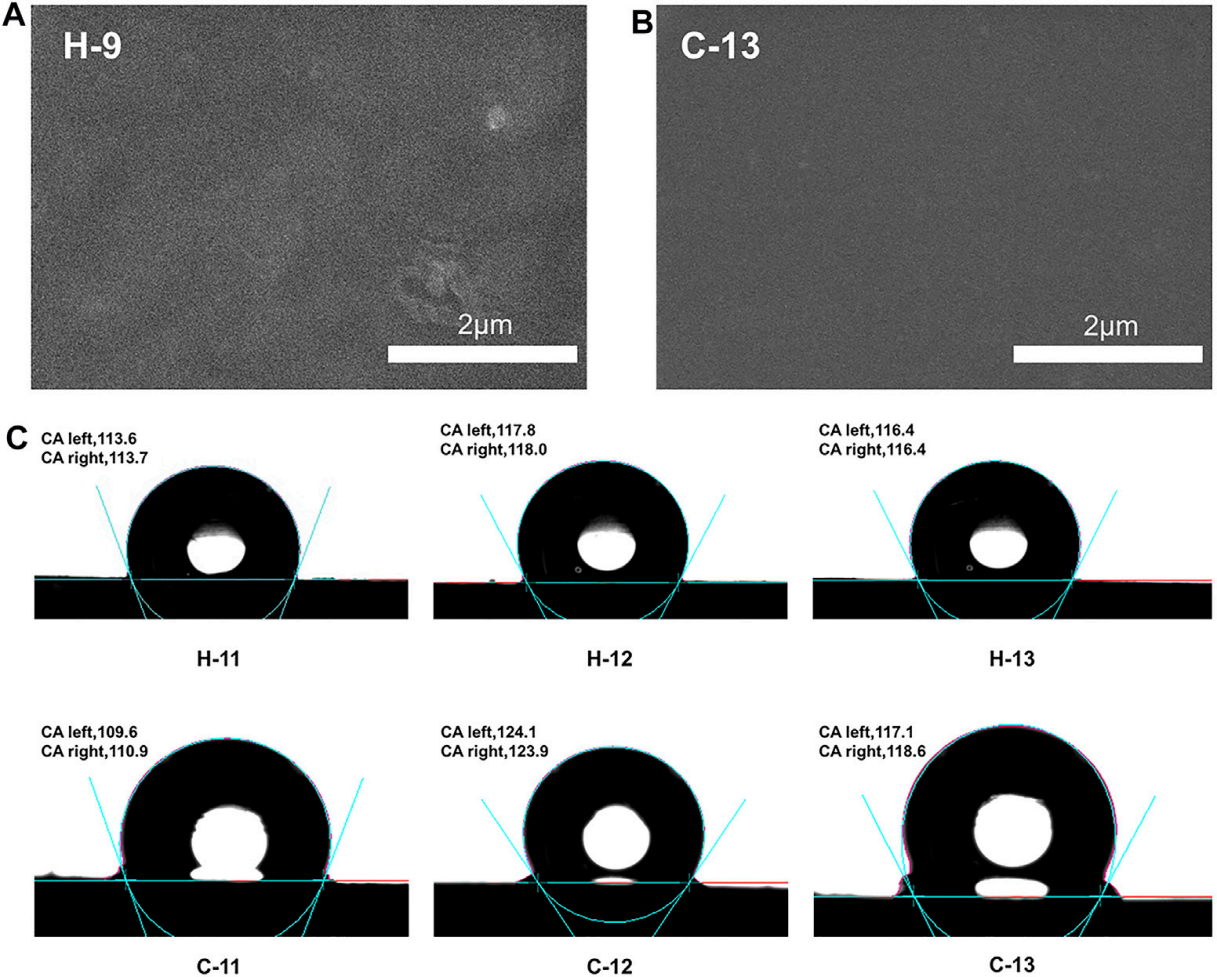
**(A)** SEM image of H-9; **(B)** SEM image of C-13; **(C)** the contact angles of silicone elastomers, H-11 to H-13 and C-11 to C-13.

The micromorphology of these SEs were observed by SEM technique. H-9 and C-13 were selected as examples. It was found that they display uniform surface morphology (Figures 10A,B), confirming that the hydrogen bond crosslinking and covalent bond crosslinking have occurred and the system is not a simple physical mixture. The hydrophobic properties of these elastomers were estimated by measuring the static contact angles. As shown in Figure 10C, these elastomers exhibit similar hydrophobicity compared to traditional SEs. The maximum contact angle of hydrogen bond crosslinked SEs is about 118° (H-11 to H-13 as examples), while that of covalent bond crosslinked SEs is about 124° (C-11 to C-13 as examples). These results indicate the good hydrophobicity of these elastomers.

### Thermal Stability

**FIGURE 11:**
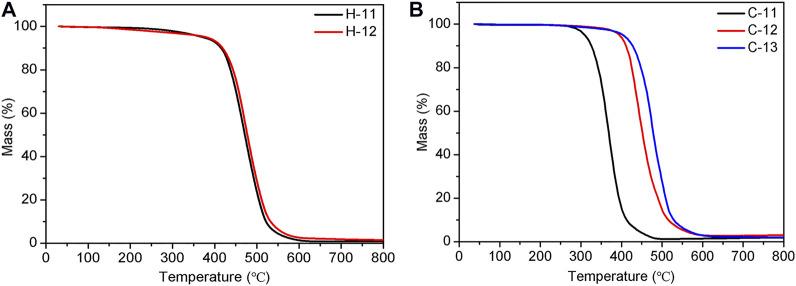
TGA curves of hydrogen bond crosslinked SEs, H-11 and H-12 **(A)**, and covalent bond crosslinked SEs, C-11 to C-13 **(B)**.

The thermal stability of these elastomers was also investigated by thermogravimetric analysis (TGA) because the decomposition temperature influences their applications. The experiments were conducted at a heating rate of 10 K/min under the atmosphere of nitrogen. Figure 11 depicts the TGA curves of two types of crosslinked SEs (H-11, H-12, and C-11 to C-13 as examples). The results reveal that the TA dosage for the hydrogen bond crosslinked does not have a significant effect on thermal stability and the SEs exhibit a high thermal stability with the T_d, 5%_ (5% weight loss temperature) at 372.5°C (Figure 11A). This finding can be explained by the reversible feature of hydrogen bonding, while the thermal stability is mainly contributed by the polysiloxane chains. In comparison, the increment of TA dosage leads to a higher thermal stability for the covalent bond crosslinked SEs with the T_d, 5%_ from 308.6°C (C-11) to 401.8°C (C-13) (Figure 11B). This finding could be attributed to improved crosslinked density with an increment of TA dosage.

### Application as Adhesives

**FIGURE 12:**
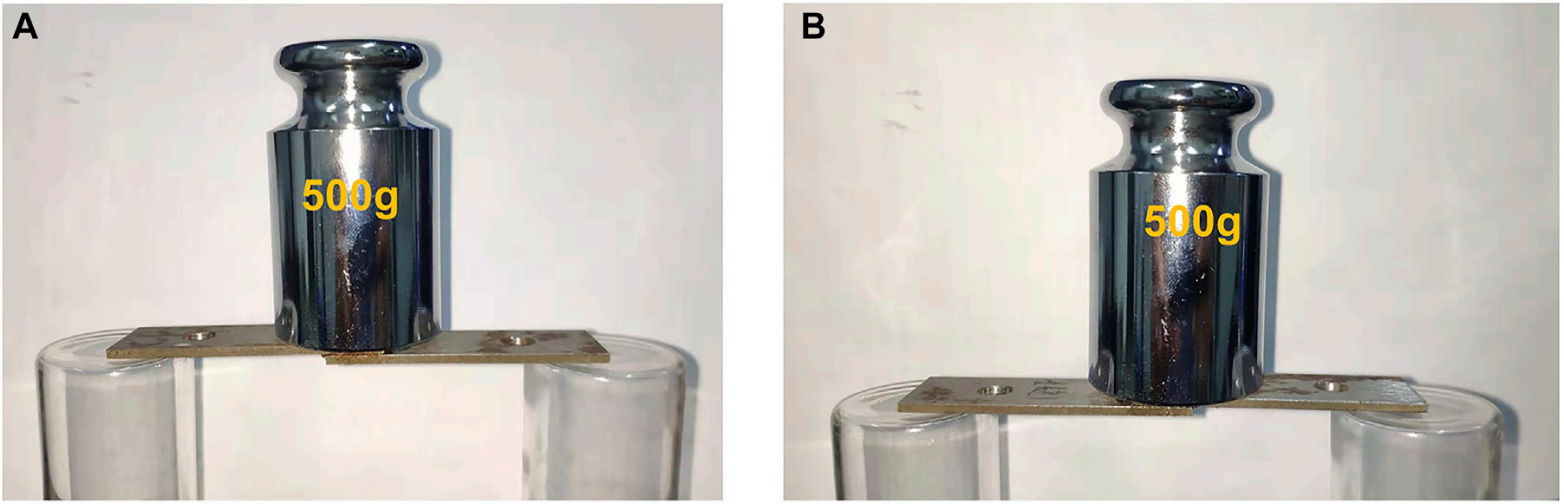
Images of adhesive performance test bonding by hydrogen bonding SEs **(A)** and covalent bonding SEs **(B)**.

To explore the potential applications of these materials, the elastomers were used as adhesives. The adhesive property was conducted by bonding two iron sheet together with a lap joint. The formulations for bonding are summarized in Supplementary Table S4. The results revealed that the prepared specimens are fully capable of carrying 500 g of weight for both hydrogen bond crosslinking and covalent bond crosslinking (Figure 12). In addition, the effect of amino content on the adhesive property was also investigated. It was found that the shear strength of both hydrogen bond crosslinking and covalent bond crosslinking increase with an increment of amino content, and the highest shear strength is 0.40 MPa (Supplementary Table S5).

## Conclusion

Novel silicone elastomers have been prepared using amino-containing polysiloxanes as the base polymers and tannic acid as a natural crosslinker under a catalyst-free method. By altering the reaction conditions, the silicone elastomers have been successfully crosslinked by hydrogen bond and covalent bond. The formability and mechanical properties of these silicone elastomers can be tuned by processing technique, the amount of TA and aminopropyl-terminated polydimethylsiloxane, the molecular weight and -NH_2_ content of PAPMS, and the amount of reinforcing filler. The hydrogen bonding mechanism can be proved by the reversible crosslinking feature of the elastomers, which can be gradually dissolved in THF and re-formed after removing THF. The covalent bond crosslinking contains the simultaneous occurrence of hydroxylamine reaction and Michael addition reaction, evidenced by a model reaction of catechol and *n*-decylamine. These elastomers exhibit good thermal stability and excellent hydrophobic property and can be applied as adhesives for bonding iron sheets to hold the weight of 500 g. To our best of knowledge, this report provides the first example of silicone elastomers crosslinked by tannic acid under a catalyst-free method. In contrast to conventional silicone crosslinking technologies, the present strategy does not require any catalyst and the crosslinker is from nature, which can meet the requirement of green chemistry. By virtue of large database and low-cost of natural polyphenols, more organic elastomers and other crosslinked materials can be readily prepared by selecting amine-containing polymers and using the present tannic acid and other polyphenols as natural crosslinkers and thus their extensive applications could be promisingly explored.

## Data Availability

The original contributions presented in the study are included in the article/Supplementary Material, further inquiries can be directed to the corresponding author.
