# Supplementary material for: Tannic Acid as a Natural Crosslinker for Catalyst-Free Silicone Elastomers From Hydrogen Bonding to Covalent Bonding
Source: Front Chem. 2021 Oct 18;9:778896. doi: 10.3389/fchem.2021.778896 (PMC8558560; doi:10.3389/fchem.2021.778896)
Supplement: Supplementary file 1 [file DataSheet1.docx]

Supplementary Material

Tannic Acid as a Natural Crosslinker for Catalyst-Free Silicone Elastomers from Hydrogen Bonding to Covalent Bonding

Sen Kong ^1^, Rui Wang ^1^, Shengyu Feng ^1,2^, Dengxu Wang^1,2^*

^1^ National Engineering Research Center for Colloidal Materials & Key Laboratory of Special Functional Aggregated Materials, Ministry of Education, School of Chemistry and Chemical Engineering, Shandong University, Jinan 250100, P. R. China

^2^Shandong Key Laboratory of Advanced Organosilicon Materials and Technologies & State Key Laboratory of Fluorinated Functional Membrane Materials, Zibo 256401, P. R. China

*** Correspondence:**Dengxu Wang
dxwang@sdu.edu.cn

# Characterization

^1^H NMR and ^13^C NMR were performed on a Bruker AVANCE 400 spectrometer at 25℃ using CDCl_3_ or DMSO-*d*_6_ as the solvent and without tetramethylsilane as an interior label. Fourier transform infrared spectra (FT-IR) were conducted on a Bruker TENSOR27 infrared spectrophotometer using the KBr pellet technique and recorded in the range of 4000-400 cm^-1^ with 4 cm^-1^ resolution and 16 scans. Gel permeation chromatography (GPC) measurements were performed on a Waters 515 liquid chromatograph (Milford, MA) equipped with a refractive-index detector 2414. Samples were run in THF at 40°C at a rate of 1 mL min^-1^. The thermal stability was performed with a Mettler Model SDTA-854 TGA Thermogravimetric Analyzer in the range of 30~800 ℃ under nitrogen with a flow rate of 100 mL/min while the heating rate was 10 ℃/min. The contact angle was conducted on a Data physics OCA-20 contact angle analyzer using distilled water as the test liquid. The hardness was measured on Shore A durometer (LaizhouHuayin Research Instruments Co., China) using ASTM D 2240 method. The tensile properties of elastomers were tested using the 3340 model Instron universal testing machine at room temperature with a test speed of 100 mm/min.

# Supplementary Figures


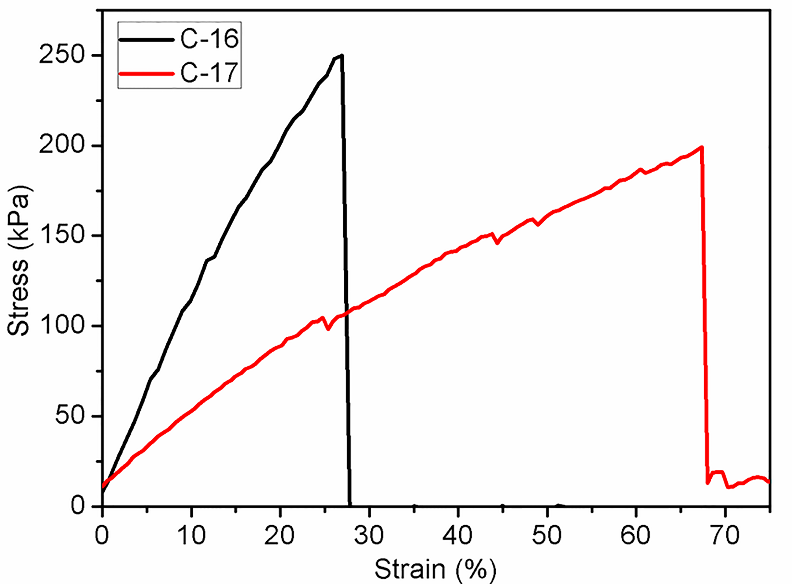


**FIGURE S1** Tensile curves of covalent cross-linked silicone elastomers (C-16 and C-17) influenced by molecular weight


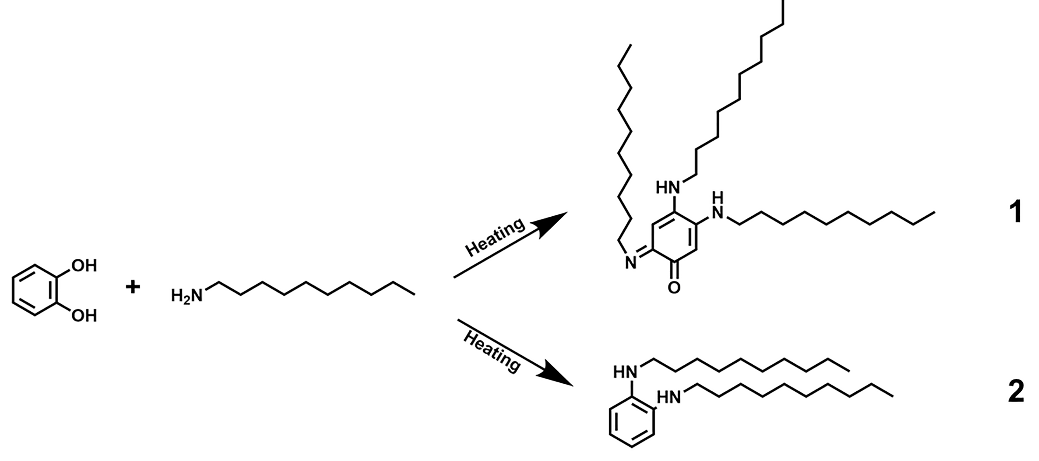


**FIGURE S2.** Possible products of the model reaction


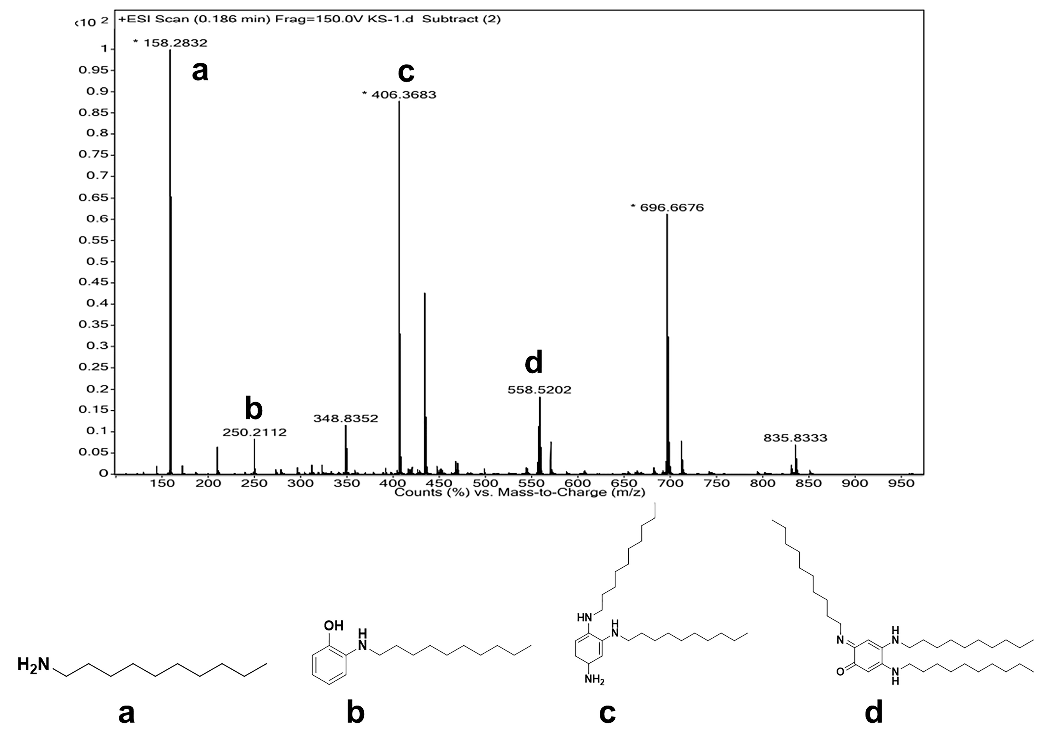


**FIGURE S3** HR-MS spectra of MC. a, b, c and d are the components in MC.


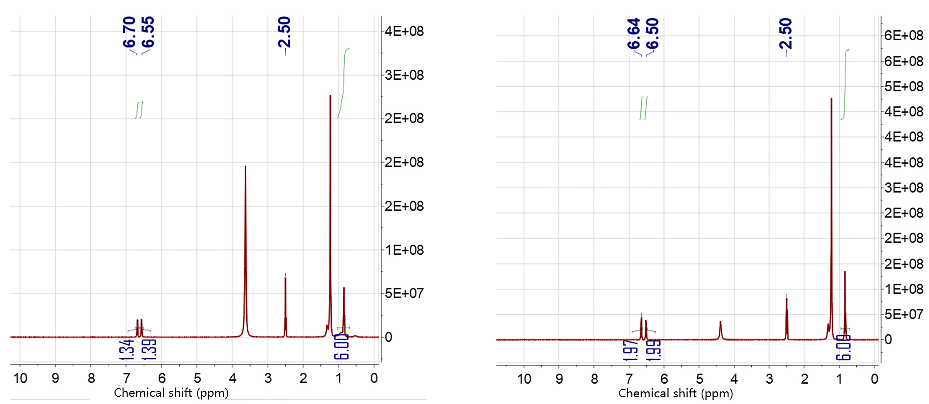


**FIGURE S4** (left) ^1^H NMR (DMSO-*d*_6_) of MC; (right) ^1^H NMR spectra (DMSO-*d*_6_) of the mixture of catechol and *n*-decylamine before the reaction.

# Supplementary Tables

**TABLE S1** Data of covalent bond cross-linked silicone elastomers

| Entry | PAPMS (g) | DP-1(g) | TA(mg) | | Organic solvents /H_2_O (mL) | 150℃ heating time (h) | | Sample description |
| --- | --- | --- | --- | --- | --- | --- | --- | --- |
| Heating time | | | | | | | | |
| C-1 | P-4.8 (1.6) | 0.6 | 30 | HEX (15/0.2) | | 1 | Formable | |
| C-2 | P-4.8 (1.6) | 0.6 | 30 | HEX (15/0.2) | | 2 | Formable | |
| C-3 | P-4.8 (1.6) | 0.6 | 30 | HEX (15/0.2) | | 3 | Formable | |
| C-4 | P-4.8 (1.6) | 0.6 | 30 | HEX (15/0.2) | | 4 | Formable | |
| DP-1 dosage | | | | | | | | |
| C-5 | P-2.5(1.6) | 0 | 60 | HEX (15/0.3) | | 1 | Formable | |
| C-6 | P-2.5(1.6) | 0.6 | 60 | HEX (15/0.3) | | 1 | Formable | |
| -NH_2_ molar content | | | | | | | | |
| C-7 | P-2.5(1.6) | 0.6 | 60 | HEX (15/0.3) | | 1 | Formable | |
| C-8 | P-5.8(1.6) | 0.6 | 60 | HEX (15/0.3) | | 1 | Formable | |
| C-9 | P-7.5(1.6) | 0.6 | 60 | HEX (15/0.3) | | 1 | Formable | |
| TA dosage | | | | | | | | |
| C-10 | P-5.8 (1.6) | 0.6 | 8 | HEX (15/0.2) | | 1 | × | |
| C-11 | P-5.8 (1.6) | 0.6 | 10 | HEX (15/0.2) | | 1 | Formable | |
| C-12 | P-5.8 (1.6) | 0.6 | 30 | HEX (15/0.3) | | 1 | Formable | |
| C-13 | P-5.8 (1.6) | 0.6 | 60 | HEX (15/0.3) | | 1 | Formable | |
| C-14 | P-5.8 (1.6) | 0.6 | 90 | HEX (15/0.4) | | 1 | Formable | |
| C-15 | P-5.8 (1.6) | 0.6 | 120 | HEX (15/0.4) | | 1 | × | |
| Molecular weight change (P-2.2: M_n_=163305，P-2.5: M_n_=64000) | | | | | | | | |
| C-16 | P-2.5 (1.6) | 0.6 | 30 | HEX (15/0.2) | | 1 | Formable | |
| C-17 | P-2.2 (1.6) | 0.6 | 30 | HEX (15/0.2) | | 1 | Formable | |

**TABLE S2** Formulation data for hydrogen bonding and covalent crosslinking with the addition of H2000

| Entry | P-2.5 (g) | | DP-1  (g) | | TA(mg) | | HEX / H_2_O (mL) | | | H2000  (g) | 150℃ heating time | | Sample description |  |
| --- | --- | --- | --- | --- | --- | --- | --- | --- | --- | --- | --- | --- | --- | --- |
| Hydrogen bonding crosslinking system | | | | | | | | | | | | | |  |
| H-17 | 1.6 | 0.6 | | | 30 | 20/0.2 | | | 0.22 | | \ | | Formable |  |
| H-18 | 1.6 | 0.6 | | | 30 | 20/0.2 | | | 0.33 | | \ | | Formable |  |
| H-19 | 1.6 | 0.6 | | | 30 | 20/0.2 | | | 0.44 | | \ | | Formable |  |
| H-20 | 1.6 | 0.6 | | | 30 | 20/0.2 | | | 0.88 | | \ | | Formable |  |
| H-21 | 1.6 | 0.6 | | | 30 | 20/0.2 | | | 1.1 | | \ | | × |  |
| Covalent cross-linking system | | | | | | | | | | | | | | |
| C-18 | 1.6 | 0.6 | | 30 | | | 20/0.2 | 0.22 | | | 1h | Formable | |  |
| C-19 | 1.6 | 0.6 | | 30 | | | 20/0.2 | 0.33 | | | 1h | Formable | |  |
| C-20 | 1.6 | 0.6 | | 30 | | | 20/0.2 | 0.44 | | | 1h | Formable | |  |
| C-21 | 1.6 | 0.6 | | 30 | | | 20/0.2 | 0.88 | | | 1h | Formable | |  |

**TABLE S3** Mechanical properties of elastomers with H2000

| Entry | Tensile strength  (MPa) | | Elongation at break  (%) | Young's modulus (MPa) | Crosslink density (mol/L) |
| --- | --- | --- | --- | --- | --- |
| H-17 | 0.5 | | 58 | 1.3 | 0.27 |
| H-18 | 0.9 | | 82 | 2.2 | 0.25 |
| H-19 | 0.8 | | 203 | 1.7 | 0.32 |
| H-20 | 1.9 | | 111 | 7.4 | 0.45 |
| C-18 | 1.0 | | 47 | 2.8 | 0.29 |
| C-19 | 1.4 | | 56 | 3.4 | 0.57 |
| C-20 | 1.5 | | 40 | 5.5 | 0.75 |
| C-21 | | 3.0 | 37 | 13.2 | 1.23 |

**TABLE S4** Binder formulation

| Entry | PAPMS(g) | DP-1(g) | TA (mg) | HEX / H_2_O (mL) | 150℃ heating time |
| --- | --- | --- | --- | --- | --- |
| Hydrogen bonding crosslinking system | | | | | |
| B-1 | P-2.5(1.6) | 0.6 | 30 | 5/0.2 | / |
| B-2 | P-4.8(1.6) | 0.6 | 30 | 5/0.2 | / |
| B-3 | P-5.8(1.6) | 0.6 | 30 | 5/0.2 | / |

Covalent cross-linking system requires heating at 150°C for 1 h.

**TABLE S5.** Binder shear strength data

| Entry | B-1 | B-2 | B-3 |
| --- | --- | --- | --- |
| Hydrogen bonding crosslinking | 0.24 MPa | 0.25 MPa | 0.33 MPa |
| Covalent bond crosslinking | 0.27 MPa | 0.23 MPa | 0.40 MPa |
